# EFPC: An Environmentally Friendly Power Control Scheme for Underwater Sensor Networks

**DOI:** 10.3390/s151129107

**Published:** 2015-11-17

**Authors:** Qiuling Yang, Yishan Su, Zhigang Jin, Guidan Yao

**Affiliations:** 1 School of Electronic and Information Engineering, Tianjin University, Tianjin 300072, China; E-Mails: yql0515@163.com (Q.Y.); qingniao0125@163.com (G.Y.); 2 College of Information Science & Technology, Hainan University, Haikou 570228, China

**Keywords:** UWSNs, marine mammals, power control, passive localization

## Abstract

In oceans, the limited acoustic spectrum resource is heavily shared by marine mammals and manmade systems including underwater sensor networks. In order to limit the negative impact of acoustic signal on marine mammals, we propose an environmentally friendly power control (EFPC) scheme for underwater sensor networks. EFPC allocates transmission power of sensor nodes with a consideration of the existence of marine mammals. By applying a Nash Equilibrium based utility function with a set of limitations to optimize transmission power, the proposed power control algorithm can conduct parallel transmissions to improve the network’s goodput, while avoiding interference with marine mammals. Additionally, to localize marine mammals, which is a prerequisite of EFPC, we propose a novel passive hyperboloid localization algorithm (PHLA). PHLA passively localize marine mammals with the help of the acoustic characteristic of these targets. Simulation results show that PHLA can localize most of the target with a relatively small localization error and EFPC can achieve a close goodput performance compared with an existing power control algorithm while avoiding interfering with marine mammals.

## 1. Introduction

Under Water Sensor Networks (UWSNs) have become a very active research area during the past decade because of their wide applications including scientific/commercial exploration, disaster prediction, environmental monitoring and oceanography data collection [1,2,3]. All these applications have motivated research on UWSN design.

As for long-range wireless communication underwater, acoustic communication is considered to be efficient [4]. However, the low attenuation of acoustic signal leads to inefficient spatial reuse, which ultimately decreases the throughput of underwater sensor networks. In [5], the authors concluded that, compared with radio based network, the throughput of UWSNs can be highly decreased because of the lower attenuation characteristic of acoustic signal. The reason is that lower attenuation signal can enlarge the interference area and decrease the spatial reuse efficiency. Spatial reuse efficiency is defined as the number of parallel transmissions, which can be conducted in one network. With less number of parallel transmissions, the throughput of the network is decreased.

In order to reduce the interference among sensors and improve the goodput of one network, power control is considered to be an efficient method. The aim of power control is to better reuse spatial resource and save power. Therefore, power control schemes for wireless networks can be generally classified into two classes: energy oriented [6,7] and throughput oriented [8,9]. Many existing power control schemes dedicated to UWSNs [10,11,12] aim to save network energy. However, these approaches may not maximize the network throughput, which is one of the most important metric of network performance. To improve the system’s performance, UPC-MAC (Underwater Power Control MAC), which is proposed in [13], leverages transmission power and long propagation delay to enhance spatial reuse efficiency.

**Figure 1:**
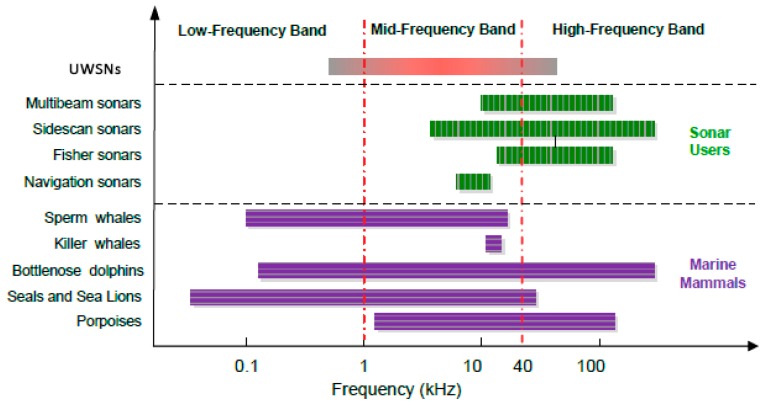
Spectrum share in underwater [15].

However, to date, most research on power control for UWSNs only focuses on the single network scenario. Nevertheless, in oceans, there exist both natural acoustic systems, for example marine mammals, and artificial acoustic systems such as sonar systems and UWSNs. These acoustic systems might share the same water area using sound for communication, sensing, detection or navigation. Unfortunately, due to the frequency-dependent attenuation feature underwater, the available communication frequency is usually from tens of hertz to hundreds of kilo hertz [14], which has been heavily shared by all existing underwater acoustic systems, as shown in Figure 1.

To efficiently utilize the spectrum resource while avoiding negative interference with other acoustic systems, especially marine mammals, smart UWSNs should be aware of surrounding environment and be able to reconfigure their operation parameters. In [15], the authors advocated cognitive acoustic (CA) as a promising technique to develop environmentally friendly UWSNs.

Furthermore, many studies have illustrated that anthropogenic noise including sonar, ship and acoustic communication can affect marine mammals by causing hearing injuries, masking of biological sounds or behavioral responses [16,17]. Jepson *et al.* [18] investigated the reasons for fourteen beaked whales being stranded in the Canary Islands, Spain, 2002, which is close to the site of an international naval exercise (Neo Tapon, 2002). They presented evidence of acute and chronic tissue damage in stranded cetaceans that resulted from the formation *in vivo* of gas bubbles. The incidence of such cases during a naval sonar exercise indicates that acoustic factors could be important in the aetiology of bubble-related disease.

Therefore, the impact on marine mammals should be taken into consideration when we develop underwater communication systems.

In this paper, we propose an environmentally friendly power control (EFPC) scheme. The proposed scheme aims to better reuse spatial resource while avoiding negative impact of acoustic signal on marine mammals by controlling transmission power. In order to limit the impact on marine mammals, the knowledge of their positions is a prerequisite. However, most of the existing localization schemes, which are designed to localize nodes in artificial systems, cannot be applied directly to localize marine mammals. The cross-correlation based methods such as [19,20] are some important methods that show good performance for marine mammals’ localization. However, the cross-correlation method needs synchronization at sample level between receivers and the transfer of a large amount of data between the nodes (or to a master node). This is hard to implement in real systems due to the energy and computation limitations. Therefore, to solve this problem, we propose a simple localization method in this paper that reduces data transmission among nodes and does not require stringent synchronization. We first propose a novel passive hyperboloid localization algorithm (PHLA). PHLA is a distance measuring based localization algorithm that does not need to known the sending time or the sending power level of the acoustic signal produced by marine mammals. Based on the acoustic characteristic of marine mammals, PHLA searches possible sending power levels of the signal, and then verify all these potential sending power levels to find the most likely position with a series of criterions. With the known position of marine mammals, EFPC can allocate the transmission power level of sensor nodes. Specifically, working with the reservation MAC protocol, EF-MAC, sensor nodes collect channel gains between senders and receivers in a distributed manner. With the gathered channel’s attenuation information, EFPC formulates an optimum problem. The objective of such problem is to maximize the goodput of the network while preventing interference with the detected marine mammals. Finally, by solving a N.E. (Nash Equilibrium) equation, each sensor’s optimal sending power is acquired.

The rest of the paper is organized as follows. In Section 2, we briefly introduce the acoustic characteristic of marine mammals. Then we present our novel passive localization scheme and power control algorithm in Section 3. Section 4 evaluates the performance of PHLA and EFPC. Finally, we draw conclusions in Section 5.

## 2. Impact of Anthropogenic Noise on Marine Mammals

To illustrate our motivation of the research on environmentally friendly power control scheme, we will first introduce the acoustic characteristic of marine mammals in this section. For most of marine mammals, sound is the main tool to collect environmental information, either through active echolocation or passive listening. However, these marine mammals, having a good sense of hearing, are vulnerable to human disturbance. Due to a mechanical coupling between the swim bladder and hearing organ, many species of marine mammals have optimum hearing sensitivity from several Kilohertz to a hundred Kilohertz [16]. 

From [16], we can observe that the frequency band on which marine mammals are most sensitive is the band used for underwater acoustic communication (1–40 KHz). Furthermore, many studies on sonar systems with the operating frequency of 1–10 KHz have demonstrated that such signal can affect marine mammals by changing their normal behaviors [16].

To prevent injury on marine animals by exposing them to anthropogenic noise, National Marine Fisheries Services (NMFS) has developed guidance on sound characteristic that are likely to cause damage and behavioral disruption (Table 1), where PTS is permanent threshold shift and TTS is temporary threshold shift.

Based on the guidance, we should develop a power control scheme for UWSNs that can avoid causing injury to marine mammals by underwater sensor nodes. The scheme will be introduced in the next section.

**Table 1:** National Oceanic and Atmospheric Administration fisheries current in-water acoustic thresholds [21].

Criterion	Criterion Deﬁnition	Threshold
Level A	PTS (injury) conservatively based on TTS	180 dB re µPa *a*
Level B	Behavioral disruption for impulsive noise	160 dB re µPa *a*

## 3. EFPC Power Control Scheme

**Figure 2:**
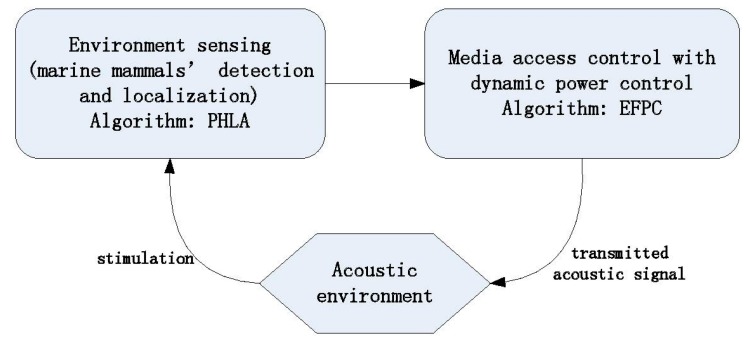
Power control framework.

Considering underwater communication systems can affect marine mammals, we develop a framework of environmentally friendly power control scheme involving two components, namely environment sensing and Medium Access Control (MAC) with dynamic power control, as shown in Figure 2. For the environment sensing component, it is mainly responsible for marine mammals’ detection and localization with our proposed passive localization algorithm: PHLA. For the MAC with dynamic power control component, it is used for scheduling packet transmission with our proposed power control scheme: EFPC. The transmission power level of sensor nodes should be limited by the sound threshold guidance shown in Table 1. To better protect marine mammals, we adopt level B (160 dB re µPa) as the threshold of received power level in our algorithm design. This means that the proposed power control scheme should limit the received power level of marine mammals bellowing below this threshold.

### 3.1. Power Control Scheme Overview

We assume that there are four anchor nodes which know their positions with existing localization techniques [22]. Each of them is within the maximum transmission range of others in order to exchange message. Sensor nodes are randomly deployed in a three-dimension area.

The purpose of our proposed Environmentally Friendly Power Control algorithm (EFPC) is to limit the negative effect of acoustic signal produced by underwater sensor nodes on marine mammals. Therefore, the knowledge of marine mammals’ position is a prerequisite. To detect and localize marine mammals, anchor nodes cooperate in a detection mode periodically and synchronically with the help of some existing synchronization algorithms, such as [23,24]. If an anchor node operates in the detection mode, it will listen to a specific channel that is used for marine mammal communication. Once anchor nodes receive a signal that is produced by marine mammals, they will run our proposed Passive Hyperboloid Localization Algorithm (PHLA) to localize these marine mammals. After that, the position information will be broadcasted to other sensor nodes in the network. With the position information of these marine mammals, EFPC can allow sensor nodes to transmit messages efficiently with a consideration of limiting the impact on the marine mammals.

### 3.2. Environmentally Friendly Power Control Scheme

With the known position of marine mammals, underwater sensor nodes can allocate their transmission power with the EFPC power control algorithm. Specifically, based on the known distance between senders and marine mammals, sensor nodes can calculate the corresponding signal attenuation from senders to marine mammals, which is the key factor for power control. The goals of EFPC are three folds: maximize network throughput, reduce energy consumption and avoid interfering with marine mammals. In order to improve network’s throughput, EFPC should efficiently reuse space resource by allowing for as many concurrent transmissions as possible. However, scheduling concurrent transmission relies on sending request collection among sensor nodes, which is a great challenge for underwater sensor networks without central nodes. To solve this problem, a related MAC protocol, named Environment-Friendly MAC (EF-MAC), dedicated to underwater sensor network was proposed by us previously. By working combined with EF-MAC, EFPC can adjust transmission power in a distributed manner.

#### 3.2.1. EF-MAC

**Figure 3:**
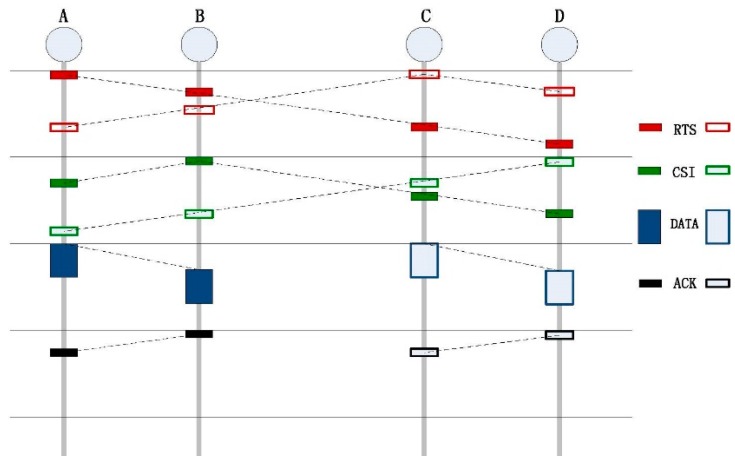
Environmentally Friendly Medium Access Control (EF-MAC) workflow.

Environmentally Friendly Medium Access Control (EF-MAC) is a kind of reservation MAC protocol that employs slotted division in the time scheduling. Based on the exchanging of control packets, EF-MAC can gather nodes’ sending request and attenuation information between theses receivers and senders. With the collected information, sensor nodes can schedule their transmission, and then allocate the transmission power by EFPC. The overall work flow of the EF-MAC combined with EFPC can be explained by an example, as shown in Figure 3.

In Figure 3, four nodes, which are within the same collision domain, are deployed in the network. Here, *TYPE_sender−receiver_* is used to denote a packet and *h_sender receiver_* to represent the channel gain. *TYPE* can be one of *RTS*, *CSI*, *DATA or ACK*. For example, *A* and *C* request to send a packet with destinations to *B* and *D*, respectively.

In EF-MAC, in order to avoid the impact of long propagation delay of acoustic signal and save energy, time is divided into slots. The detail design of the length of a slot can be found in [25]. The length of one slot is τ+γ+α, where τ is the maximum propagation delay of transmitted signal, and γ is the transmission time of one *CSI* (Channel State Information) packet. To compensate clock drift in the system, α, the length of guard time, is applied. When a node needs to send packets, it waits until the end of the present slot and then senses the channel. Once the channel is detected as idle, the node will send an *RTS* packet at the beginning of next slot (defined as slot 1). In this example, nodes *A* and *C* send *RTS_A−B_* and *RTS_C−D_* with the maximum power level to nodes *B* and *D*. Both of the destinations will receive these two packets. Each *RTS* contains two elements: the packet’s priority and the level of sending power. The packet’s priority, which is set by sensor nodes, is related to the packet’s generation time. The earlier the packet is generated, the higher priority it has. Then, with the receiving and original sending power levels of one packet, node *B* obtains channel gain: *h_AB_*, *h_CB_* with Equation (1) and node *D* can calculate the channel gain: *h_AD_*, *h_CD_*:

(1) $$h(dB)=p_{r}(dB)-p_{s}(dB)$$

where *h* denotes channel gain in *dB*, $p_{r}$ denotes receiving power and $p_{s}$ denotes the transmission power.

In this paper, Thorp attenuation model, which can be replaced by any other more realistic and advanced models (e.g., raytracing or normal modes), is applied as attenuation model. In those cases, since *A(d)* will not be in general monotonic, the solution of the distance may be more difficult. 

Nodes *B* and *D* will schedule *CSI* packets at the beginning of the next slot (slot 2). The *CSI* packets contain the channel gain, which is calculated in the previous slot (slot 1). Thus, at the end of slot 2, *A* and *C* construct a matrix of channel state, named H. Every sender node will construct an *N* *× N* of H as Equation (2), where N is the number of sending nodes:

(2) $$H=\left[\begin{array}{l} h_{AB}\;h_{AD}\\ h_{CB}\;h_{CD} \end{array}\right]$$

After that, with the channel state matrix, every sender allocates it sending power of *DATA* in a distributed manner at the beginning of slot 3 by the power control scheme named EFPC. At the beginning of slot 4, *B* and *D* will schedule ACK (Acknowledgment) packet if they correctly receive *DATA* previously. Otherwise, they need to back off a random number of slots to retransmit the *RTS*.

The basic idea of the proposed MAC scheme is that the sending request from one sender should be jointly considered with the request from other senders. With such MAC scheme and related power control scheme (EFPC), many parallel communication can be accomplished in the network.

#### 3.2.2. Power Control Algorithm: EFPC

Based on the channel state matrix and given positions of marine mammals (if there is any), sensor nodes can calculate their proper transmission power for *DATA* packet’s transmission. With a consideration of marine mammals that may not be always around the network, we apply a two-mode power control scheme including a share mode and an exclusive mode.

##### Share Mode

If the network has detected that there are some marine mammals, sensor nodes in the network will switch to the share mode to control their transmission power. In this mode, when a node requests sending a packet, its transmission power should not be higher than the threshold that can affect the behavior of marine mammals. In this paper, we adopt TTS (160 dB re µPa in Table 1) as the threshold.

In networks, each node is considered to be selfish but rational, which means that they want to maximize their own interest. Therefore, the transmission power allocation problem can be treated as a game that can be solved by game theory. To fulfill the goals of EFPC, a utility function that contains both utility term and pricing term is defined as Equation (3). We assume that every node shares the same bandwidth *B*. Based on this assumption, *B* is omitted when we construct the utility function. The first part of the utility function denoting the relationship between the sending power level and corresponding link’s capacity is based on Shannon theory [26]. As for the second part of the utility function, it reflects the price of consuming a certain power budget:

(3) $$\;u_{i}(p_{\;i},{\text{P}}_{-\text{i}})=log(1+\frac{h_{i}\cdot p_{i}}{\sum_{j=1}^{N-1}\;h_{j}\cdot p_{j}+\sigma^{2}})-{\text{α}}_{i}\cdot p_{i}$$

where $p_{i}$ is transmission power on the link i, $h_{i}$ is the channel gain in channel i, ${\text{P}}_{-\text{i}}$ is transmission power on other links except link i, and ${\text{α}}_{i}$ [bit/Watt], is the bit price for one unit transmission power. $\sigma^{2}$ denotes noise power (variance). The noise model presented in [27] is applied.

Finally, the power allocation problem is formulated as Equation (4) and substitution is in Equation (5):

(4) $$max\;u_{i}(p_{\;i},{\text{P}}_{-\text{i}})=log(1+\frac{h_{i}\cdot p_{i}}{\sum_{j=1}^{N-1}\;h_{j}\cdot p_{j}+\sigma^{2}})-{\text{α}}_{i}\cdot p_{i}$$

(5) $$\begin{array}{l} s.t.\\ p_{\;i}\in \left[0,\;P_{max}\right]\;(1\leq i\leq N)\\ \frac{h_{i}\cdot p_{i}}{\sum_{j=1}^{N-1}\;h_{j}\cdot p_{j}+\sigma^{2}}\geq S\;I\;N\;R_{th}\;(1\leq i\leq N)\\ \sum_{i=1}^{N}h_{mi}\cdot p_{i}\leq T \end{array}$$

where *P_max_* is the maximum sending power limited by hardware design, $S\;I\;N\;R_{th}\;$ is the decoding threshold of acoustic modem, *T* is the behavioral interruption threshold (TTS), and *h_mi_* is the channel gain between sender and marine mammals. The third constraint denotes that the receiving power level from all senders should not be larger than the behavioral interruption threshold (TTS).

The optimal power allocation problem can be solved by Nash Equilibrium (N.E.). Before we solve the problem, the existence of N.E. of this problem should be proved. As stated in [26], N.E. exists only if the following two conditions are satisfied:

1. $S_{i}$, the set of transmission power of every sending node, is a convex subset of Euclidean space.
2. $u_{i}$, for every sending node, is a continuous and quasi-concave function for variable *p_i_*.

Condition 1 is obviously satisfied. Therefore, we will prove condition 2 is satisfied next. The second-order derivation of $u_{i}$ is:

(6) $$\frac{\partial^{2}u_{i}}{\partial p_{i}^{2}}=-\frac{h_{i}^{2}}{{\left(h_{i}\cdot p_{i}+\sum_{j=1}^{N-1}\;h_{j}\cdot p_{j}+\sigma^{2}\right)}^{2}}$$

Since $\frac{\partial^{2}u_{i}}{\partial p_{i}^{2}}$ < 0, we can conclude that condition 2 is also satisfied. Therefore the existence of N.E. is proven.

With the game theory, the optimal transmission power of each sender can be seemed as the players’ response function, which is defined as Equation (7).

(7) $$\frac{\partial u_{i}}{\partial p_{i}}=-\frac{h_{i}}{\left(h_{i}\cdot p_{i}+\sum_{j=1}^{N-1}\;h_{j}\cdot p_{j}+\sigma^{\;2}\right)}-\text{α}=0$$

Solving Equation (7) gives:

(8) $$p_{i}=\frac{1}{{\text{α}}_{i}}-\frac{\sum_{j=1}^{N-1}\;h_{j}\cdot p_{j}+\sigma^{\;2}}{\;h_{i}}$$

In order to guarantee $p_{i}\leq P_{max}$, we choose $\text{α}{\;}_{i}=\frac{1}{P_{max}}$.

For all the sending nodes, after rearranging Equation (8), we can derive Equation (9):

(9) $$P^{*}=H^{-\;1}\;\cdot \;G$$

H is a square matrix; $P^{*}$ is the unique N.E. solution; and *G* = [*g*_1_, *g*_2_*...g_n_*]^*T*^ is an *N* *×* 1 vector, where *g_i_* = $\frac{h_{i}}{{\text{α}}_{i}}-\sigma^{\;2}$.

If the final $p_{i}$ cannot satisfy constraint 3, all the senders, except the one with lowest priority of sending request, will back to the power allocation algorithm.

After $P^{*}$ is derived, we should check every entry of the matrix to make sure all the three limitations are satisfied. If some of these limitations are not satisfied, the sensor node with highest priority of its request can only be allowed to transmit data. The transmission power can be calculated by Equation (10):

(10) $$p_{i}=T/h_{mi}$$

##### Exclusive Mode

If the network does not detect any marine mammal, sensor nodes can transmit their packets in an exclusive mode. In this mode, the aim of EFPC is to maximum the network throughput while reducing energy consumption. Thus, we can formulate the optimization problem as Equation (11) with two limitations.

(11) $$max\;u_{i}(p_{\;i},{\text{P}}_{-\text{i}})=log(1+\frac{h_{i}\cdot p_{i}}{\sum_{j=1}^{N-1}\;h_{j}\cdot p_{j}+\sigma^{2}})-{\text{α}}_{i}\cdot p_{i}$$

(12) $$\begin{array}{l} s.t.\\ p_{\;i}\in \left[0,\;P_{max}\right]\;(1\leq i\leq N)\\ \frac{h_{i}\cdot p_{i}}{\sum_{j=1}^{N-1}\;h_{j}\cdot p_{j}+\sigma^{2}}\geq S\;I\;N\;R_{th}\;(1\leq i\leq N) \end{array}$$

The only difference compared with share mode is that the third constraint, which is used for limiting the impact on marine mammals, is deleted. Based on the similar deduction, we can also allocate the transmission power by Equation (13):

(13) $$P^{*}=H^{-\;1}\;\cdot \;G$$

where H is a square matrix; $P^{*}$ is the unique N.E. solution; and *G* = [*g*_1_*, g*_2_*...g_n_*]*^T^* is an *N* *×* 1 vector, where *g_i_* = $\frac{h_{i}}{{\text{α}}_{i}}-\sigma^{\;2}$.

Similar to the share mode, if some of these limitations are not satisfied, the sensor node with highest priority of its request can be allowed to transmit data. However, in this mode, it can transmit the data packet with a full power.

### 3.3. PHLA: Passive Hyperboloid Localization Algorithm

**Figure 4:**
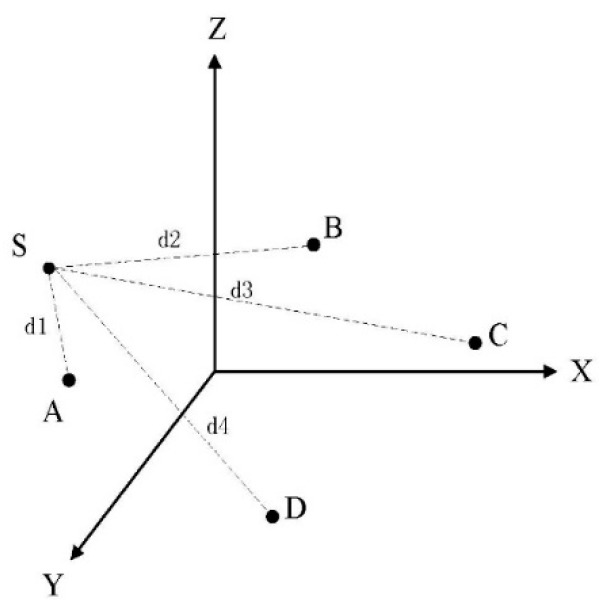
Passive Hyperboloid Localization Algorithm (PHLA) algorithm.

We will present our novel passive hyperboloid localization algorithm (PHLA) in this sub-section. PHLA is a passive localization scheme, which is based on distance measuring. The basic idea for a localization algorithm with distance measuring is that if the distances between a target (marine mammal) and the anchors are given, then the position of the target can be solved. For an instance, as shown in Figure 4, S is the target to be localized and A, B, C, D are four anchor nodes with known positions. If *d*1*−d*4 are given, the position of S can be solved by a spherical localization or a hyperboloid localization algorithm [28,29]. To estimate the distance of *d*, methods such as Time of Arrival (TOA) [30] and Received Signal Strength Indicator (RSSI) [31] have been proposed. However, none of these approaches can be applied for marine mammals localization directly. The reason is that these approaches measure distance relying on the known sending time or sending power level of the signal. However, none of these parameters can be obtained directly from marine mammals. To solve this problem, we propose a searching based RSSI algorithm to localize the target passively. A searching based RSSI algorithm searches possible sending power levels of the target in a finite interval, and then verifies these values to find the most likely power level.

PHLA is a three phase scheme including signal detection, message exchange and localization. In signal detection phase, anchor nodes detect a specific frequency channel, which is related to the hardware design of acoustic modem and strength of particular acoustic signal produced by marine mammals, for example the signal of dolphins [32]. Signal detection and classification of marine mammals is critical for PHLA. Fortunately, many studies have been study in this field [33,34]. Therefore, in this paper, we assume that the detected signal has been classified.

If anchor nodes have detected the signal, they will switch to the message exchange phase. In this phase, each anchor node broadcast a packet that carries the frequency and strength of the signal it received and the position of itself. These parameters are used for distance’s calculation, which will be discussed later. Finally, with the information of the signal, each anchor nodes estimate the position of marine mammals by PHLA algorithm in a distributed manner in the localization phase.

Here we use an example to illustrate PHLA in detail. For example, we suppose the target is a dolphin. Base on the acoustic characteristic of dolphins, the transmission power of their communication signal ranges from 150 dB re µPa to 170 dB re µPa [32], which can be transferred to power level as 0.01–1 W. Therefore, anchor nodes can search possible transmission power levels with a step (e.g., 0.01 W) from 0.01 W to 1 W. 

In our localization algorithm, we first consider range of the source, says 0.01 W to 1 W, which means that the actual source level should value between 0.01 W and 1 W. Then the anchor nodes try to search the right source level with a step (e.g., 0.01 W). For every tested source level (the tested source level is denoted as *Ps* in Equation (14)), the anchor nodes can obtain a corresponding distance between anchor and target based on Equations (14) and (15):

(14) $$10log\frac{P_{s}}{P_{r}}=A(d,f)$$

(15) $$A(d,f)=10k\cdot logd+\frac{d}{1000}\cdot 10log\text{α}(f)$$

where *d* is the distance between the target and anchors, $P_{s}$ is the tested transmission power level, $P_{r}$ is the received power level, *A* is the channel gain, *k* is the spreading factor with a typical value 1.5 in underwater [35,36], *f* is the frequency of the acoustic signal, and α is the absorption factor.

Since the tested source level may be or may not be the real source level, we should verify the tested source level by following steps:

Once the distances between the target and anchor nodes are given, for any two of the distance, for example *d*_1_ and *d*_2_ in Figure 4, in order to set up a triangle, they should be first verified by Equation (16):

(16) $$d_{1}+d_{2}>d_{AB}$$

Only the qualified transmission power level will be used for localization next. With the distance information, we can apply hyperboloid localization algorithm to localize the target [27,37]. The basic idea of hyperboloid localization method is to utilize the difference of the distance from target to reference nodes (anchor nodes) to establish a hyperboloid formula. Then, the focus of three hyperboloids is the position of the target. For example, as in Figure 4, $d_{i1}=d_{i}-d_{1}\;(i=2,3,4)$ are known. For each $d_{i1}\;(i=2,3,4)$, we can set up a hyperboloid formula, and then calculate the position of the focus. The related process can be founded in [29]. After hyperboloid localization process, three circumstances may exist:

1. Only one $P_{S}$ and a corresponding position $P(x_{s},y_{s},z_{s})$ exist. This value can be concluded as the position of the target;
2. None of $P_{S}$ exists. This may result from the blind area in hyperboloid localization, which will be discussed later. The PHLA algorithm failure;
3. More than one $P_{S}$ exists. Then a best qualified node is selected based on Equations (17)–(21).

(17) $$\left|\sqrt{{\left(x_{s}-x_{a}\right)}^{2}+{\left(y_{s}-y_{a}\right)}^{2}+{\left(z_{s}-z_{a}\right)}^{2}}-d_{1}\right|={\text{ε}}_{a}$$

(18) $$\left|\sqrt{{\left(x_{s}-x_{b}\right)}^{2}+{\left(y_{s}-y_{b}\right)}^{2}+{\left(z_{s}-z_{b}\right)}^{2}}-d_{2}\right|={\text{ε}}_{b}$$

(19) $$\left|\sqrt{{\left(x_{s}-x_{c}\right)}^{2}+{\left(y_{s}-y_{c}\right)}^{2}+{\left(z_{s}-z_{c}\right)}^{2}}-d_{3}\right|={\text{ε}}_{c}$$

(20) $$\left|\sqrt{{\left(x_{s}-x_{d}\right)}^{2}+{\left(y_{s}-y_{d}\right)}^{2}+{\left(z_{s}-z_{d}\right)}^{2}}-d_{4}\right|={\text{ε}}_{d}$$

(21) $$\text{ε}={\left({\text{ε}}_{a}^{2}+{\text{ε}}_{b}^{2}+{\text{ε}}_{c}^{2}+{\text{ε}}_{d}^{2}\right)}^{\frac{1}{2}}$$

where $x_{s},y_{s},z_{s}$ is the potential positions of the target, $x_{a},y_{a},z_{a};\;x_{b},y_{b},z_{b};\;x_{c},y_{c},z_{c}$ are the position of anchor A, B, C, respectively. The potential position with the minimum ε is selected, which means that the selected position is the one with minimum localization error.

**Figure 5:**
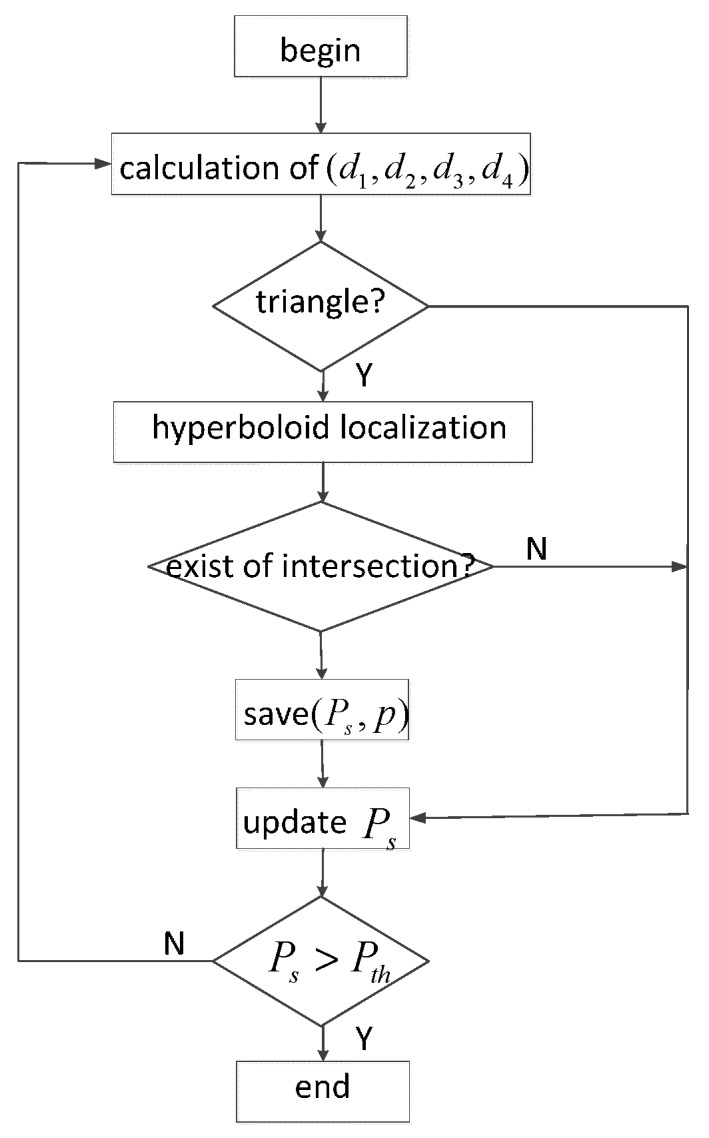
PHLA work flow.

The work flow of PHLA is shown in Figure 5, where *p* is the potential position of target and $P_{th}$ is the maximum threshold of received power level. The outputs of this work flow are some qualified position. From these potential positions, the one with minimum ε is considered as the position of the target.

## 4. Evaluation

In this section, we will first evaluate PHLA algorithm in terms of localization precision and success ratio, which is defined as the ratio of the number of points that are correctly localized to the total number of points that need to be localized. Furthermore, we evaluate the performance of a network that applies EFPC algorithm combined with EF-MAC to control transmission power. As a comparison, we also evaluate the performance of SFAMA, a classic slotted based MAC protocol without power control [25] and EF-MAC, respectively. In this section, we will evaluate (a) the goodput of the network, which is defined as the number of bits of successful transmitted data in unit time; and (b) end-to-end delay, which is defined as the average end-to-end delay from source to destination.

The simulations of EFPC protocols are evaluated by an NS2 based simulator: Aqua-sim [38]. Some modules in physical layer are modified to adjust transmission power level. We adopt the hardware parameters from real implemented underwater OFDM modems [39], which are listed in Table 2.

**Table 2:** Simulation parameters.

Maximum Transmission Range	3000 m
Simulation time	10^4^ s
Full power	40 W

To evaluate both localization algorithms and MAC protocols in a more realistic underwater environment, the characteristic of underwater channel is obtained by Bellhop model, which accurately models the underwater sound propagation by a specific ray tracing algorithm. The Bellhop model provides an accurate model of the propagation of acoustic signal in ocean environments for a good characterization of the signal propagation behavior.

**Figure 6:**
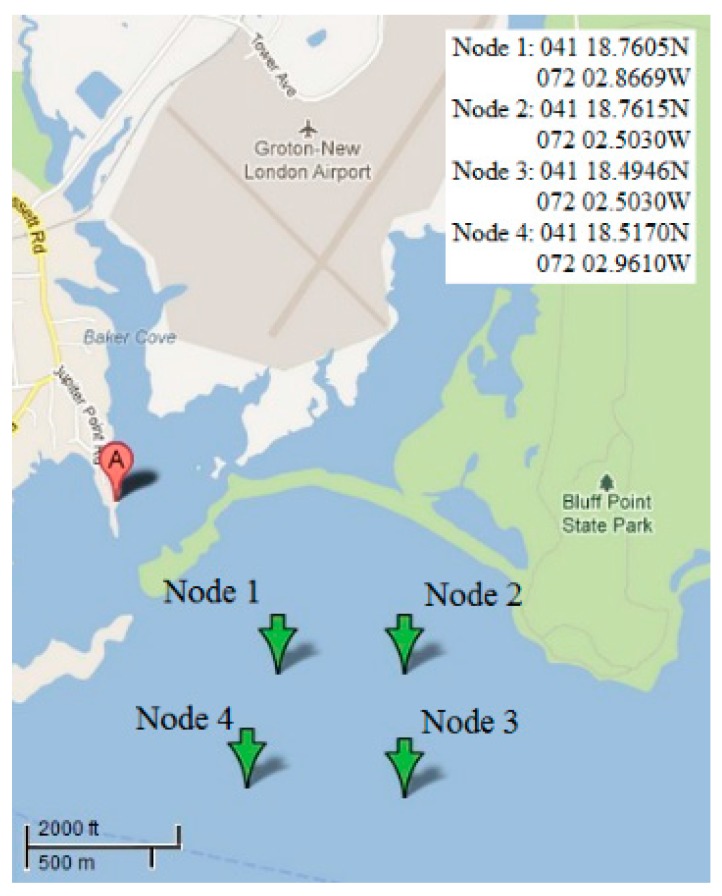
Area of collected channel data.

**Figure 7:**
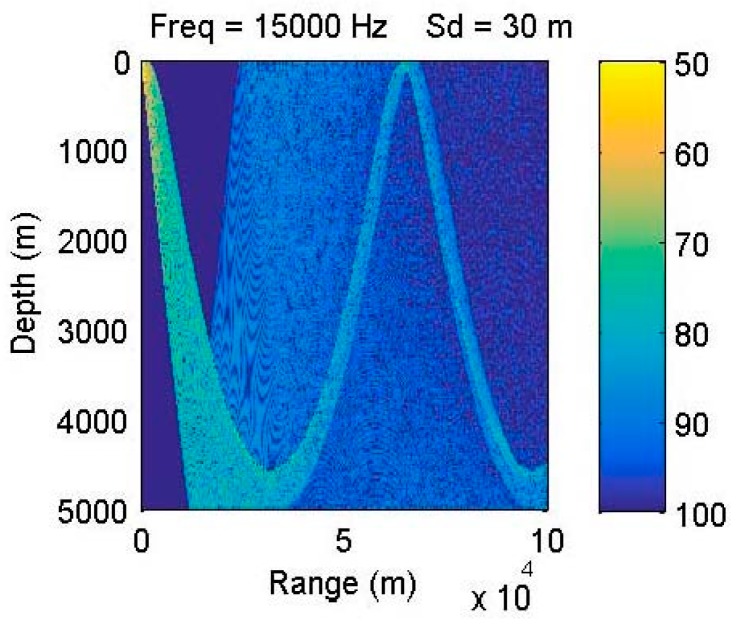
Transmission loss in the selected area.

Real environmental data are used, from a deployed UAN testbed named Ocean-TUNE [40]. It locates in the Atlantic Ocean off the coast of the Long Island Sound (CT, USA) (Figure 6). The simulation parameters set in Bellhop are as follow: bottom reflection coefficient is 1; the surface reflection coefficient is −1. The depth of four anchor nodes is set to be 30 m below the water surface. The depth of water is 50 m. Sound speed profiles (SSP), bathymetry profiles and information on the type of bottom sediments of the selected area are obtained from the World Ocean Database [41], from the General Bathymetric Chart of the Oceans (GEBCO) [42] and from the National Geophysical Data Center’s Deck41 database [43], respectively. With Bellhop model, the channel attenuation feature is shown in Figure 7.

### 4.1. PHLA Evaluation

**Figure 8:**
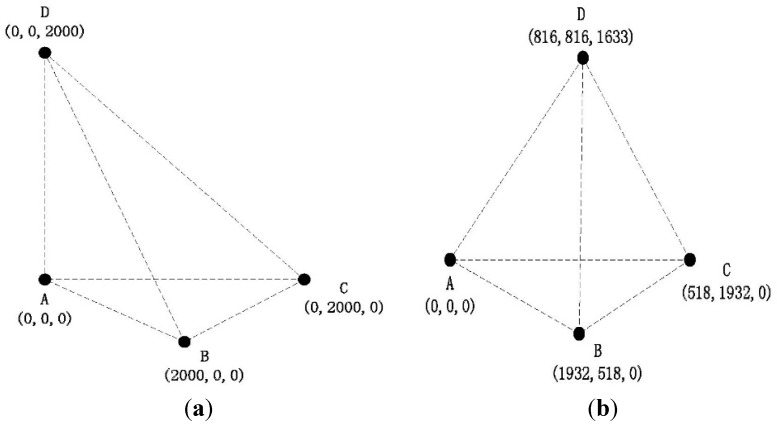
Anchors’ deployment: (**a**) Senario 1; and (**b**) Scenario 2 (normal triangular pyramid).

To evaluate the impact of different anchor’s deployment schemes on the PHLA algorithm, we evaluate the following two scenarios as show in Figure 8. In each scenario, 1000 points are selected randomly in the localization area as potential positions of marine mammals. By comparing the outcomes of the estimation with the real ones, we can evaluate the performance. In both scenarios, the distance between anchors is fixed to 2000 m. We assume that the frequency and SPL (sound pressure level) of acoustic signal produced by marine mammals are 15 kilo Hz and 160 dB re µPa, respectively [32].

As we know, the frequency of the received signal is related with the velocity of marine mammals due to the Doppler effect, which may further influence the performance of the localization algorithm. In order to evaluate this impact, we take into account sixteen different velocities including four different directions, each of which has four different magnitudes. The directions are [1,1,0], [−1,−1,0], [1,0,0], and [−1,0,0], which are denoted by ***v***_1_, ***v***_2_, ***v***_3_, and ***v***_4_, respectively. The reasons why we choose these four directions are that most of time marine mammals swim at the same depth and the projection of reference nodes are symmetric about the line y = x in plane. Besides, the four magnitudes of velocity are 5 m/s, 10 m/s, 15 m/s, and 20 m/s in reference to the speed range of marine mammals [44]. 

#### 4.1.1. Success Rate of Localization

Table 3 illustrates the success rate of localization with different velocities in scenario 1. We show the results for scenario 1 and 2 in this paper. As shown in Table 3, it is obvious that both the magnitude and direction of velocity of the marine mammal have effect on the success rate of localization, but there is no regular relationship between the magnitude of velocity and the success rate. This is because the success rate of localization depends on the error of distance estimation, which is mainly determined by velocity. However, in the proposed localization algorithm, we substitute the average frequency of received signals for the real transmission frequency of the signal. When the magnitude of velocity increases, the absolute frequency offset of all received signals increases with different extents, whether it is negative or positive, which leads to the uncertainty of change trend of average frequency.

**Table 3:** Success rate of localization with different velocities in scenario 1 (2).

Velocity	*v*_1_	*v*_2_	*v*_3_	*v*_4_
5 m/s	0.918 (0.893)	0.897 (0.889)	0.917 (0.891)	0.917 (0.890)
10 m/s	0.901 (0.878)	0.899 (0.873)	0.889 (0.875)	0.897 (0.876)
15 m/s	0.896 (0.873)	0.875 (0.860)	0.897 (0.858)	0.893 (0.851)
20 m/s	0.916 (0.884)	0.879 (0.861)	0.907 (0.870)	0.870 (0.862)

Due to the discussion above, we introduce average success rate of localization, the mean of success rates of localization with different velocities in a certain scenario to analyze the approximate influence of deployment on the performance of localization. As shown in the Table 4, we can get that the deployment of anchor nodes does affect success rate of localization.

**Table 4:** Average success rate of localization in different scenarios.

Deployment	Average Success Rate of Localization
Scenario 1	89.8%
Scenario 2	87.4%

**Figure 9:**
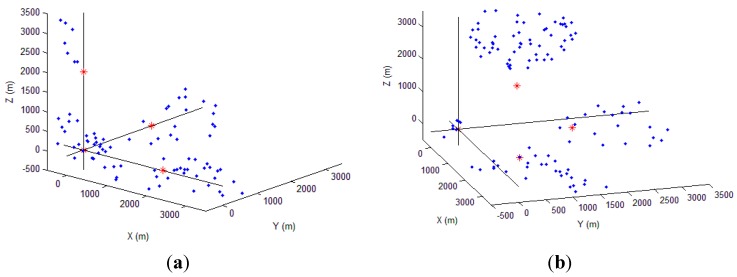
Blind areas: (**a**) scenario 1 and (**b**) scenario 2.

Furthermore, Figure 9 shows the blind area of two scenarios. The four red points denotes anchor nodes with known positions. The positions marked by blue dots are the points that cannot be correctly localized by PHLA. We can observe that most of the un-localized positions locate around anchor nodes. The reason is that hyperboloid localization method localizes a target based on the focus of three hyperboloids. However, a target near the anchor nodes may lead to large curvature on some of the hyperboloids. In that case, a tiny error, which results from the step searching scheme in PHLA, on distance measurement will result in a failure of the localization. After collecting statistical results, the success ratio of localization is 89.8% and 87.4%, respectively, for two different scenarios, which means PHLA can cover most of the area in both scenarios.

#### 4.1.2. Accuracy of PHLA Localization Algorithm

Localization accuracy is another significant metric of a localization algorithm. From the deduction of PHLA, we can find that a known frequency of the transmission signal is a prerequisite for distance measurement from Equations (14) and (15). However, due to the Doppler effect, which results from the movement of marine mammals, the frequency of the received signal may not be coincident among anchor nodes. As a result, the distance measurement may be imprecise. Therefore, we evaluate the relationship between the speed of marine mammal’s movement and the localization error, which is defined as Equation (22):

(22) $$e_{v}=\frac{1}{N}\sum_{1}^{N}\left|s_{i,v}-s_{i,v}^{*}\right|$$

where $s_{i,v}$ and $s_{i,v}^{*}$ are the accurate position and estimated position, respectively, and *N* is the number of total localized positions.

**Figure 10:**
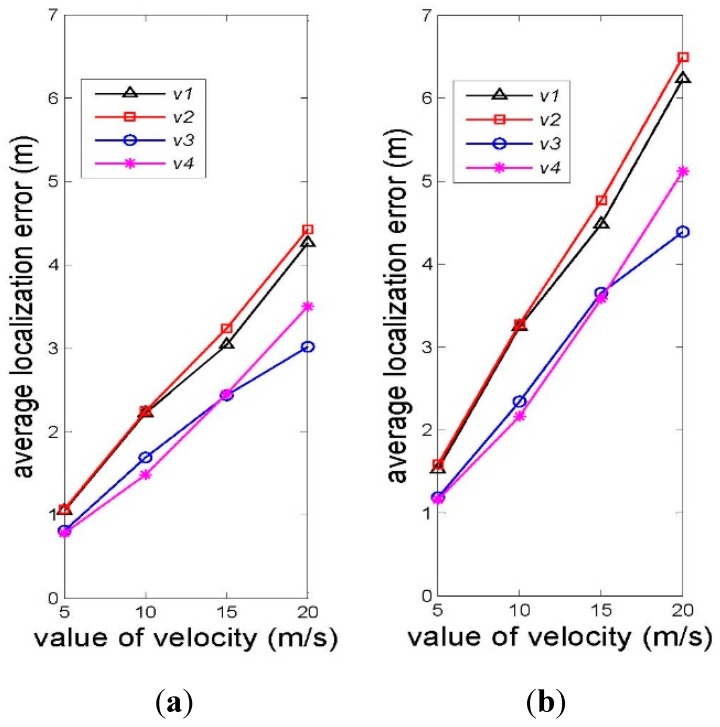
Average localization error *vs.* value of velocity: (**a**) scenario 1 and (**b**) scenario 2.

Figure 10 illustrates the average localization error with varying the velocity of marine mammals in different scenarios. Generally, a similar variation trend is observed in the three scenarios that average localization error increases as the magnitude of velocity increases. This is because the increase in the magnitude of velocity enlarges the frequency offset of received signals, which finally causes the increase in the error of distance estimation. Additionally, the direction of velocity has an impact on the localization performance and the two opposite directions make little difference, as the gap between the green line and the red one corresponding with ***v***_1_ and ***v***_2_ show. This is because the direction of the velocity affects velocity component on the line determined by the reference node and the position of the marine mammal, the essential factor to frequency offset of the received signal. Since the velocities with the opposite directions have the same magnitude, the only difference of the velocity component is the direction, whose influence is relatively small.

For both of the metric’s evaluations, we observe that PHLA can localize marine mammals with a relatively high success ratio and accuracy.

### 4.2. EFPC Evaluation

**Figure 11:**
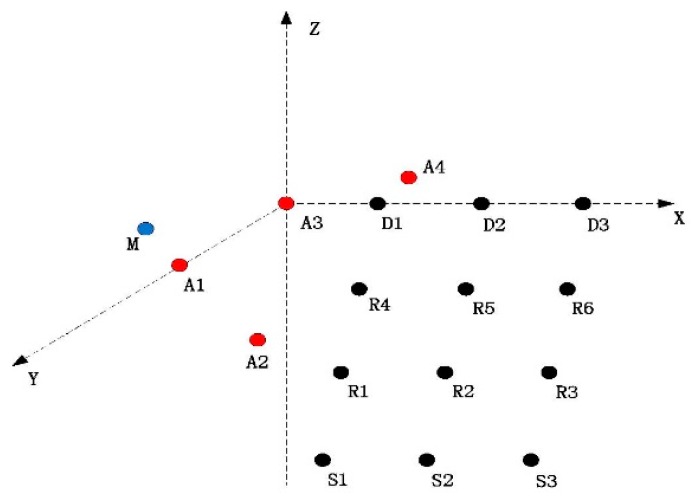
Network topology.

The topology of the network is shown in Figure 11. A1–A4 are four anchor nodes, which are used for localizing sensor nodes and marine mammals. Sinks D1–D3 locate on the water surface and are the destinations of data packets. Other nodes are deployed in a three-dimensional area. In the network, we assume that S1–S3 are data sources and R1–R6 are relay nodes. Each sensor node is deployed in the middle of a field with a size 1 km × 1 km × 1 km. We assume a marine mammal, *M*, moves with a speed, **v**.

**Figure 12:**
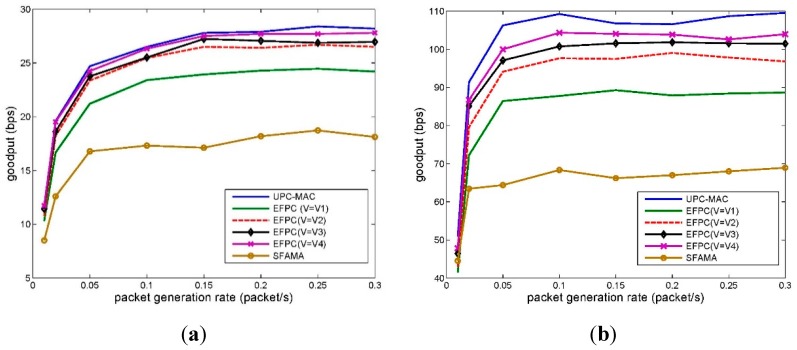
Network goodput. (**a**) Packet length 100 B; (**b**) Packet length 500 B.

To evaluate the impact of **v** on the performance of the network, we evaluate the goodput performance with a set of **v** as Table 5. Figure 12a depicts the network goodput with varying speed **v** of marine mammal represented by *M*.

**Table 5:** Velocity setting.

v1	(1, 0, 0)
v2	(2 ,0, 0)
v3	(0, 1, 0)
v4	(0, 2, 0)

This figure shows that the existence of marine mammals does lower the goodput of the network. The reason is that, by employing EFPC, if the network has detected that there are marine mammals, sensor nodes will switch to the share mode. In share mode, some of the potential communication between sensor nodes may be postponed or prevented if the received power level by marine mammals goes beyond the threshold.

However, even for the worst case (**v** = **v_1_**), the goodput of the network can still achieve 88% of that in EF-MAC, which does not consider the existence of marine mammals. There are two main factors attribute to the result. The first reason is that only parts of the network may be affected by the marine mammals. Many of the sensor nodes can still schedule their transmission by EFPC algorithm. The second reason is that moving marine mammals will not always be affected by the network. For example, even for the slowest speed **v_1_**, *M* will go across the network in about 5000 s, which is 50% of the total simulation time.

Furthermore, compared with SFAMA, EFPC can improve the goodput of the network by about 33%–53% for different velocity settings. The reason is that the power control scheme can efficiently reuse the spatial resource by allowing for as much parallel transmissions as possible.

By comparing the goodput performance among different sets of **v**, we observe that both the value and the direction of velocity can affect the performance of the network. For the same direction, the faster the M moves, the higher the goodput the network can achieve. This is because that a faster speed reduces the time when *M* is affected by the network. With the same reason, the direction of velocity can also affect the goodput of the network. For example, with **v_2_**, node *M* goes through the network with a less time compared with **v_1_**, which results a less reduction of the goodput of the network.

For a longer packet scenario, 500 B in Figure 12b, the curves have similar trends to Figure 12a. Furthermore, the goodput of network has about five times better achievement compared to Figure 12a. The reason is mainly because that EFPC works combined with a slotted based MAC protocol: EF-MAC. For EF-MAC, both 100 B and 500 B packets can be conducted in one slot. The length of packet has little impact on the transmission scheduling in MAC. Therefore, if we double the packet’s length, the goodput of the network will be doubled. Compared with SFAMA, the improvement of goodput performance for the longer packet length setting is about 30%–51%.

**Figure 13:**
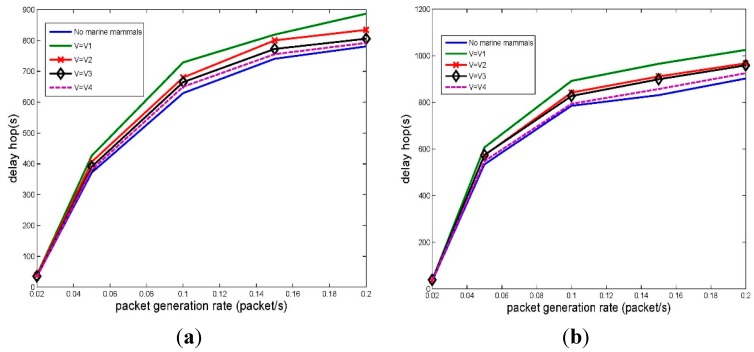
Network delay. (**a**) Packet length 100 B; (**b**) Packet length 500 B.

The end-to-end delay is also important metric of network; therefore, we also compare it with the set of velocities shown in Table 3. Figure 13 show that the network which is affected by marine mammals leads to a higher end-to-end delay. For the same direction of velocity, the lower speed leads to a higher end-to-end delay. The reason is that a lower speed leads to a longer duration that the network is impacted by marine mammals. During that time duration, some of the packets were postponed for transmission, which increase the end-to-end delay.

## 5. Conclusions and Future Work

In this paper, we develop a novel environmentally friendly power control scheme, EFPC, and a corresponding passive localization algorithm, PHLA, for UWSNs. EFPC is a power control scheme that can switch between a share mode and an exclusive mode to tradeoff between network performance and marine mammal protection. In the share mode, EFPC can avoid negative impact on marine mammals by limiting transmission power level. To improve the network performance, if there are no marine mammals around the network, EFPC will switch to the exclusive mode. In this mode, EFPC can fully utilize spatial resource to maximize the goodput of network. Simulation results show that PHLA can localize most of the targets with a relatively small error and EFPC can achieve a close goodput performance compared to an existing power control algorithm, while avoiding interfering with marine mammals.

Regarding future works, considering that underwater channel state may change a lot during packet transmission, and the movement of marine mammals can impact on localization accuracy, a joint design of power control algorithm channel estimation and movement prediction will improve the overall performance of the network. 
